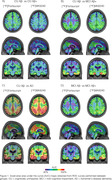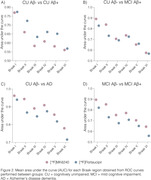# Voxel‐wise comparison of [^18^F]MK6240 and [^18^F]Flortaucipir for the diagnosis of individuals across the Alzheimer’s disease spectrum – the HEAD study

**DOI:** 10.1002/alz.092862

**Published:** 2025-01-09

**Authors:** Bruna Bellaver, Guilherme Povala, Guilherme Bauer‐Negrini, Firoza Z Lussier, Livia Amaral, Pamela C.L. Ferreira, Val J. Lowe, David N. soleimani‐meigooni, Hwamee Oh, Dana Tudorascu, William J. Jagust, William E Klunk, Belen Pascual, Brian A. Gordon, Pedro Rosa‐Neto, Suzanne L. Baker, Tharick Ali Pascoal

**Affiliations:** ^1^ University of Pittsburgh, Pittsburgh, PA USA; ^2^ Department of Radiology, Mayo Clinic, Rochester, MN USA; ^3^ Memory and Aging Center, Weill Institute for Neurosciences, University of California, San Francisco, San Francisco, CA USA; ^4^ Brown University, Providence, RI USA; ^5^ University of California, Berkeley, Berkeley, CA USA; ^6^ Houston Methodist Research Institute, Houston, TX USA; ^7^ Washington University in St. Louis School of Medicine, St. Louis, MO USA; ^8^ Translational Neuroimaging Laboratory, The McGill University Research Centre for Studies in Aging, Montréal, QC Canada; ^9^ Lawrence Berkeley National Laboratory, Berkeley, CA USA

## Abstract

**Background:**

In vivo studies using the tau PET tracers have shown high performance for the diagnosis of Alzheimer’s disease dementia and patterns of tracer uptake that resemble those observed in post‐mortem studies. However, tau tracers present distinct patterns of binding that might influence their performance in detecting AD pathology. In a head‐to‐head study, we investigated the performance of [^18^F]MK6240 and [^18^F]Flortaucipir for the diagnosis of AD.

**Method:**

We assessed 132 individuals from the HEAD study (58 CU Aβ‐, 15 CU Aβ+, 14 MCI Aβ‐, 32 MCI Aβ+ and 13 AD dementia) with Aβ‐PET, [^18^F]MK6240 and [^18^F]Flortaucipir. Voxel‐wise receiver operating characteristic curves (ROC) of the two tau tracers were used to contrast groups provided the area under the curve (AUC) for disease diagnosis or biomarkers positivity.

**Result:**

The brain maps showed numerically higher and more spread AUC for [^18^F]Flortaucipir to discriminate CU Aβ‐ from CU Aβ+ (Figure 1A). The difference between tracers’ AUC was greater in Braaks IV and V (Figure 2A), both regions that are not expected to have tau accumulation in CU Aβ+ individuals, reflecting a potential off‐target binding for [^18^F]Flortaucipir. To differentiate CU Aβ‐ from MCI Aβ+ individuals, [^18^F]MK6240 presented a numerically higher AUC than [^18^F]Flortaucipir in Braak I and II and similar AUC in other Braak regions (Figure 1B, 2B). We observed a high performance of [^18^F]MK6240 and [^18^F]Flortaucipir in differentiating AD dementia from CU Aβ‐ individuals. However, [^18^F]MK6240 exhibits a higher AUC than [^18^F]Flortaucipir in all Braak regions, especially Braak V‐VI (Figure 1C, 2C). Finally, [^18^F]MK6240 presented higher AUC in all Braak regions to discriminate MCI Aβ‐ from MCI Aβ+ individuals (Figure 1D, 2D).

**Conclusion:**

Our results indicate that [^18^F]MK6240 and [^18^F]Flortaucipir present high accuracy to discriminate AD from CU Aβ‐ individuals. However, [^18^F]MK6240 presents higher AUC to discriminate AD and MCI Aβ+ from CU Aβ‐ individuals than [^18^F]Flortaucipir. Together, our head‐to‐head study sheds light on the distinct patterns of binding for Tau‐PET tracers.